# Utilization of crop wild relatives for biotic and abiotic stress management in Indian mustard [*Brassica juncea* (L.) Czern. & Coss.]

**DOI:** 10.3389/fpls.2023.1277922

**Published:** 2023-10-25

**Authors:** Swati Verma, Namo Dubey, K. H. Singh, Nehanjali Parmar, Lal Singh, Dipika Sharma, Dipika Rana, Kalpana Thakur, Devina Vaidya, Ajay Kumar Thakur

**Affiliations:** ^1^ College of Horticulture and Forestry Thunag, Dr. Yashwant Singh Parmar University of Horticulture and Forestry Nauni, Solan, HP, India; ^2^ School of Biochemistry, Devi Ahilya University, Indore, MP, India; ^3^ ICAR-Directorate of Rapeseed-Mustard Research, Bharatpur, Rajasthan, India; ^4^ School of Biological and Environmental Sciences, Shoolini University of Biotechnology and Management Sciences, Solan, HP, India; ^5^ Regional Horticultural Research and Training Station Bajaura, Dr. Y. S. Parmar University of Horticulture and Forestry, Solan, HP, India

**Keywords:** *Brassica juncea*, crop wild relatives (CWRs), biotic stress, abiotic stress, protoplast fusion, embryo rescue, cytoplasmic male sterility

## Abstract

*Brassica juncea* (L.) Czern. & Coss. (Indian mustard) is an economically important edible oil crop. Over the years, plant breeders have developed many elite varieties of *B. juncea* with better yield traits, but research work on the introgression of stress resilience traits has largely been lagging due to scarcity of resistant donors. Crop wild relatives (CWRs) are the weedy relatives of domesticated plant species which are left unutilized in their natural habitat due to the presence of certain undesirable alleles which hamper their yield potential, and thus, their further domestication. CWRs of *B. juncea* namely include *Sinapis alba* L. (White mustard), *B. tournefortii* Gouan. (African mustard), *B. fruticulosa* Cirillo (Twiggy turnip), *Camelina sativa* L. (Gold-of-pleasure), *Diplotaxis tenuisiliqua* Delile (Wall rocket), *D. erucoides* L. (White wall rocket), *D. muralis* L. (Annual wall rocket), *Crambe abyssinica* R.E.Fr. (Abyssinian mustard), *Erucastrum gallicum* Willd. (Common dogmustard), *E. cardaminoides* Webb ex Christ (Dogmustard), *Capsella bursa*-*pastoris* L. (Shepherds purse), *Lepidium sativum* L. (Garden Cress) etc. These CWRs have withstood several regimes of biotic and abiotic stresses over the past thousands of years which led them to accumulate many useful alleles contributing in resistance against various environmental stresses. Thus, CWRs could serve as resourceful gene pools for introgression of stress resilience traits into Indian mustard. This review summarizes research work on the introgression of resistance against *Sclerotinia* stem rot (caused by *Sclerotinia sclerotiorum*), *Alternaria* blight (caused by *Alternaria brassicae*), white rust (caused by *Albugo candida*), aphid attack, drought and high temperature from CWRs into *B. juncea*. However, various pre- and post-fertilization barriers due to different ploidy levels are major stumbling blocks in the success of such programmes, therefore, we also insightfully discuss how the advances made in -omics technology could be helpful in assisting various breeding programmes aiming at improvisation of stress resilience traits in *B. juncea*.

## Introduction

Indian mustard [*Brassica juncea* (L.) Czern & Coss.] is an economically important, edible oilseed crop of Brassicaceae family belonging to the rapeseed-mustard (RM) group. *B. juncea* is cultivated in India, China, Bangladesh, Pakistan, Australia, Canada and some European countries (Choudhury et al., 2023). It is predominantly cultivated as an oilseed crop in India over an estimated area of 6.2 million hectare, contributing to >85% of RM acreage (Singh et al., 2022). Indian mustard oil is widely consumed as an edible oil. Mustard oil has a unique ratio of omega-3 and omega-6 fatty acids with many benefits related to cardiac health (Manchanda and Passi, 2016). Mustard oil is also rich in alpha-tocopherol and antioxidants offering many other additional health benefits. Besides edible purposes, mustard oil is also used in body massages for increasing muscle strength and treating skin problems, and in manufacturing processes of paints and varnishes (Thakur et al., 2020). After oil extraction, the left-out seed meal is used as a protein-rich feed for farm animals, especially poultry. The vegetative part of mustard is consumed as a very popular delicious cuisine known as ‘*Sarson Ka Saag*’ in the northern part of India (Ananthanarayan et al., 2019).

The actual yield potential of *B. juncea* can be realized upto 3500-4000 kg/ha, but despite continuous breeding efforts over a period of last four decades, the national average productivity of Indian mustard still hovers around 1400 Kg/ha (Sinha et al., 2020). The overall production and productivity of this oilseed crop is hampered by several biotic (*Alternaria* blight caused by *Sclerotinia sclerotiorum*, stem rot caused by *Alternaria brassicae*, white rust caused by *Albugo candida*, powdery mildew caused by *Erysiphe cruciferarum*, aphids) and abiotic factors (drought, high temperature stress - both at seedling stage and terminal stage, salinity and frost) (Thakur et al., 2020). While, due to taste preferences for mustard oil, an escalating per-capita consumption, and thus market demand is being constantly observed. India is importing large quantities of edible oil from different countries to meet the huge edible oil demands of its population. This is amounting to washing out a large exchequer of money (Sharma et al., 2022). It has been estimated that by 2025, the demand of edible oil in India will increase upto 34 million tonnes, and out of this, around 14 million tonnes had to be met by Indian mustard alone (Singh et al., 2022). Throughout the globe, various *Brassica* research groups are making concerted efforts for developing elite high-yielding varieties of *B. juncea* having resistance against biotic and abiotic stresses (Chauhan et al., 2011). Among different biotic stressors, except for some *Brassica* germplasm showing resistance against white rust, no other crossable germplasm sources have been identified for developing *B. juncea* varieties which could be resistant to *Alternaria* blight, stem rot and powdery mildew. Among abiotic stresses, several salinity tolerant *B. juncea* varieties including CS 52, CS 54, CS 56 and CS 58 have been released in India (Tripathi et al., 2012). However, no robust donor line has been identified to develop resistance against drought, frost and high temperature stress in *B. juncea*.

Crop wild relatives (CWRs) are the weedy, wild relatives of domesticated plants, which usually occur and are maintained mostly in their centers of origin (Kashyap et al., 2022; Jain et al., 2023). CWRs have been left unexploited in nature because of the presence of some undesirable genes/alleles contributing as yield barriers. CWRs of *B. juncea* majorly include *Sinapis alba* L. (White mustard), *B. tournefortii* Gouan. (African mustard), *B. fruticulosa* Cirillo (Twiggy turnip), *Camelina sativa* L. (Gold-of-pleasure), *Diplotaxis tenuisiliqua* Delile (Wall rocket), *D. erucoides* L. (White wall rocket), *D. muralis* L. (Annual wall rocket), *Crambe abyssinica* R.E.Fr. (Abyssinian mustard), *Erucastrum gallicum* Willd. (Common dogmustard), *E. cardaminoides* Webb ex Christ (Dog mustard), *Capsella bursa*-*pastoris* L. (Shepherds purse), *Lepidium sativum* L. (Garden Cress) etc. (Singh et al., 2021a). Over the years, these CWRs have withstood the selection pressure of various biotic and abiotic stress factors, developed the resistance mechanisms gradually and inherited it over the generations. A list of CWRs of *B. juncea*, their common name, ploidy level, genome size, chromosome number and economic importance is given in **Table 1**. The rigorous use of these CWRs as potential allele donors in conventional breeding programmes is hampered due to their different ploidy levels, or certain pre- and post-fertilization barriers. Further, the introduction of undesirable alleles from the CWRs into *B. juncea* due to linkage drag might result in heavy yield penalties (Bohra et al., 2022). In the past few decades, certain technological interventions have been devised for the introgression of resistance traits from CWRs into the cultivated varieties of *B. juncea*. These techniques include protoplast fusion, embryo rescue, repeated back-cross breeding with the recurrent parent followed by open pollination and new omics techniques. This review summarizes the efforts of various research groups in utilizing CWRs for combating the adverse effects of biotic and abiotic stresses on Indian mustard yield.

**Table 1:** List of CWRs of *Brassica juncea*, their common name, ploidy level, genome size, chromosome number and economic importance.

S.No.	CWR	Common name	Ploidy level	Genome size	Chromosome number	Economic importance	Reference
1	*Sinapis alba*	White mustard	2n	449.7 Mb	24	Resistance against *Alternaria* blight, SSR; high temperature and drought stress tolerance	Kumari et al. (2020a)
2	*Brassica tournefortii*	African mustard	2n	791 Mb	20	Tolerance to drought and heat stress, resistance against *Alternaria* and white rust	Kashyap et al. (2023)
3	*Brassica fruticulosa*	Twiggy turnip	2n	–	16	Resistance against mustard aphids, SSR, *Alternaria* blight, white rust; tolerance to drought stress	Atri et al. (2019); Rana et al. (2017); Atri et al. (2012); Kumar et al. (2011)
4	*Camelina sativa*	Gold-of-pleasure	2n	641.4 Mb	40	Resistance against *Alternaria* blight, mustard aphids, white rust; tolerance to drought stress	Purnamasari et al. (2019)
5	*Diplotaxis erucoides*	White wall rocket	2n	499 Mb	14	Resistance against *Alternaria* blight	Choudhury et al. (2023); Mehta et al. (2023); Vasupalli et al. (2017); Bhat et al. (2006)
6	*Crambe abyssinica*	Abyssinian mustard	2n	3500 Mb	90	Resistance against mustard aphids; tolerance to drought stress	Samarappuli et al. (2020)
7	*Camelina cardaminoides*	Dog mustard	2n	–	18	Resistance against SSR	Rana et al. (2019)
8	*Capsella bursa*-*pastoris*	Shepherds purse	2n	268.7 Mb	32	Resistance against SSR, *Alternaria* blight; tolerance to drought stress	Chen et al. (2007)
9	*Lepidium sativum*	Garden Cress	2n	336.5 Mb	16	Resistance against mustard aphids, white rust; tolerance to heat stress	Yadav et al. (2019)

-, not available.

## Use of CWRs for introgression of biotic stress resistance traits into *B. juncea*

Various biotic stresses adversely affect the yield of Indian mustard. In India, more than thirty diseases are known to affect mustard growth and yields (Saharan et al., 2005). Among them, few inflict serious damages upon mustard production depending upon their prevalence over vast geographical locations. Namely, stem rot, *Alternaria* leaf spot and white rust are major diseases caused by fungal pathogens which may amount to more than 90% yield losses in this oilseed crop (Saharan and Mehta, 2008; Kumar et al., 2012; Kamoun et al., 2015; Jyoti et al., 2021; Singh et al., 2021a). Besides these, parasitic feeding by aphid pest *Lipaphis erysimi* Kalt. leads to stunted growth, very less seed formation, and decreased oil content in Indian mustard. In the following section we discuss the efforts made by various research groups for introducing resistance traits from CWRs into Indian mustard.

## *Sclerotinia* stem rot resistance

White rot or stem rot caused by *S. sclerotiorum* is a serious fungal disease of Indian mustard. Due to changes in climatic conditions, *Sclerotinia* stem rot (SSR) has become a very serious disease of mustard. This necrotrophic fungus causes yield losses ranging from 5-100% in mustard production (Saharan and Mehta, 2008; Uloth et al., 2016; Sharma et al., 2018; Singh et al., 2021a). It also impacts mustard oil quality and reduces oil content (Inturrisi et al., 2021). Due to prolonged survival and broad infection ability, the management of SSR utilizing cultural and chemical practices could be less rewarding (Kamal et al., 2016; Singh et al., 2020). *S. sclerotiorum* is a broad host range phytopathogen which overpowers the plant defense mechanism through their interacting virulence factors (Rodriguez-Moreno et al., 2018). Recently, Gupta et al. (2022) reported the draft genome sequence of *S. sclerotiorum* “ESR-01”, an Indian isolate, and its secretory effector repertoire. It highlights the secretome, effector, carbohydrate active enzymes and PHI-base repertoire associated with the *S. sclerotiorum* genome. This knowledge has been quite helpful in refining the understanding of *S. sclerotiorum*-*Brassica* interaction.

Inadequate variation is found in *Brassica* germplasm for complete genetic resistance against *S. sclerotiorum* infection (Rana et al., 2017; Atri et al., 2019). The wild allies of *Brassicaceae*, viz., *B*. *fruticulosa*, *C. bursa*-*pastoris*, *D. tenuisiliqua*, *E. gallicum*, *E*. *cardaminoides* carry high levels of resistance against stem rot pathogen infection (Chen et al., 2007; Garg et al., 2010; Mei et al., 2011). Some of them are being utilized in different research programmes for transfer of SSR into *B. juncea*. In a study by Kumari et al. (2020a), two fertile and stable allohexaploid were generated by protoplast fusion between *B. juncea* and *S. alba*. These symmetric hybrids (2*n* = 60) remained stable throughout consecutive generations and displayed significant resistance against SSR (Kumari et al., 2020a). In another attempt, the SSR resistance from *B*. *fruticulosa* has been introgressed into Indian mustard (Rana et al., 2017). The developed introgressed lines (ILs) of *B*. *juncea*-*B*. *fruticulosa* had been assessed for their resistance against *Sclerotinia*. The cytogenetic characterization of 28 ILs showed substitution of predominantly terminal *B*. *fruticulosa* segments located on B-genome chromosomes (Rana et al., 2017). Resistance responses to *S*. *sclerotiorum* were repeat-evaluated and different ILs were genotyped for 202 transferable and 60 candidate gene simple sequence repeats. A total of 10 highly significant marker trait associations (MTAs) were achieved by association mapping. Selected ILs showed high levels of resistance against SSR (Rana et al., 2017). It has now become possible to exploit historical recombination events by utilizing genome-wide association studies (GWAS) to achieve improved mapping resolution (Tibbs Cortes et al., 2021). Genotyping by sequencing (GBS) of 88 ILs of *B*. *juncea*-*B*. *fruticulosa* helped in studying marker trait associations (MTA’s) and reported 49 significant SNPs corresponding to different loci on different chromosomes (Atri et al., 2019). This also helped in prediction of the candidate disease genes belonging to various protein families and developed an understanding of the immune responses against *S*. *sclerotiorum* in mustard. The generated marker datasets could be utilized for assisted transfer of introgressed resistant loci associated with SSR resistance into superior *Brassica* cultivars (Atri et al., 2019).

*E. cardaminoides*, another wild relative of *B. juncea*, is a likely source of resistance to many diseases including SSR (Gomez-Campo et al., 1999; Chandra et al., 2004). A set of *B. juncea-E. cardaminoides* ILs with genomic regions associated with SSR resistance was developed by Rana et al. (2019). The ILs had been evaluated for resistance responses against SSR over three crop seasons. Different SNPs associated with leucine rich repeat-receptor like kinases (LRR-RLK) genes, genetic factors associated with pathogen-associated molecular patterns (PAMPs) and effector-triggered immunity (ETI) belonging to three R-genes encoding toll-interleukin receptor- nucleotide-binding site- leucine-rich repeat (TIR-NBS-LRR) proteins have been identified in this study (Rana et al., 2019). These could be major contributing factors in SSR resistance responses. However, it is likely that some of these marker-trait associations may involve small SSR resistance responses from gene pool already present in Indian mustard. Significantly higher resistance in developed *B. juncea-E. cardaminoides* ILs indicated that majority of this response is due to introgression of genes from wild *E. cardaminoides* (Rana et al., 2019). Accelerated transcriptomic and genomics research on the developed *B. juncea-*CWR ILs would clarify the mechanisms underlying pathogenesis of *S*. *sclerotiorum* in *B. juncea*. Rana et al. (2019) have proposed taking up transcriptome-based research on *S*. *sclerotiorum*-*B. juncea* interaction. It would help the researchers in understanding how *S*. *sclerotiorum* infection interferes with different hormone signaling pathways to hijack *B. juncea* defense system. Further, spatial, and temporal changes in gene expression would be helpful in gaining advanced insights into events that lead to disease development and colonization of tissues by this pathogen. As proposed, use of *E. cardaminoides* specific oligo-probes for cytogenetical mapping of all the introgression sites in developed ILs would be helpful in understanding the CWR-specific contribution to *S*. *sclerotiorum* resistance in developed ILs.

## *Alternaria* blight resistance

The seed borne fungal pathogens of *Alternaria* spp. are causal agents of *Alternaria* leaf blight disease of *B. juncea*. Both, *Alternaria brassicicola* and *A. brassicae* cause *Alternaria* leaf blight (Sharma et al., 2022). Among the two, *A. brassicae* is reported to be more virulent, while, *A. brassicicola* co-inhabits the infected plant tissue (Sharma et al., 2002). An estimated crop-damage of upto 10-70% in *B. juncea* is inflicted by *Alternaria* leaf blight alone (Kumar and Kolte, 2012; Gupta et al., 2020), making it a serious disease of oilseed mustard. Disease infected plants are characterized by concentric ring spots which merge to develop big necrotic patches (Bohra et al., 2022). Susceptibility to *Alternaria* leaf blight is shown by almost all cultivated varieties of Indian mustard (Jyoti et al., 2021). Resistance to *Alternaria* blight is a polygenic character, therefore, introgression of traits through horizontal breeding could be a more appropriate way to transfer these traits into cultivated *Brassicas.* However, there is a lack of resistant crossable germplasm in cultivated *B. juncea*. Many cruciferous wild relatives are known to exhibit significant levels of resistance against *Alternaria* leaf blight. These include *B. desnottesii, Camelina sativa, Diplotaxis berthautii, D. catholica, D. cretacea, D. erucoides*, and *E. gallicum* (Sharma et al., 2002). Yet, various compatibility barriers (both pre- and post-fertilization barriers) impede the research programs aiming introgression of *Alternaria* blight resistance traits from CWRs into cultivated *B. juncea* (Vasupalli et al., 2017).

Embryo rescue techniques have been proposed as a strategy for introgression of resistant traits from CWRs into cultivated Indian mustard (Kumar et al., 2001; Bhat et al., 2006; Vasupalli et al., 2017)*. S. alba*, a wild ally of crop *Brassicas*, carries significant resistance against *Alternaria* black spot. In the past, attempts have been made for the development of symmetric somatic hybrids with Indian mustard. However, such attempts mostly resulted in production of male sterile hybrids, hybrids with variable pollen fertility and seed set, overall unstable hybrids (Gaikwad et al., 1996). *D. erucoides*, a CWR of *Brassicas*, shows high levels of resistance against the *Alternaria* blight pathogen, *A. brassicae*. Bhat et al. (2006) attempted to introgress *Alternaria* blight resistance from *D. erucoides* to *B. juncea* using *B. rapa* as bridging species. After two successive backcrossing of the progenies with the recurrent parent i.e., *B. juncea*, the BC2 interspecific hybrids were advanced to BC_2_F_7_ generation and simultaneously screened for *Alternaria* blight resistance, which resulted in some promising resistant Indian mustard lines (Bhat et al., 2006). Kumari et al. (2020b) generated *B. juncea-S. alba* hybrids through protoplast fusion. Two generated hybrids were reported to be symmetric, while, third had significant resemblance to *B. juncea* and thus was found asymmetric. The hybrids exhibited complete male and female fertile characteristics and normal back crossing progenies. High resistance to *A. brassicae* infection was observed in both (Kumari et al., 2020b).

An assessment of transcriptional activation of glucosinolate biosynthetic genes and glucosinolate accumulation in *Alternaria* resistant *D. erucoides* and susceptible *B. juncea* species has highlighted few potential candidate genes which augment resistance to *Alternaria* blight (Choudhury et al., 2022). More jasmonate defense signaling-mediated transcriptional activation of candidate secondary metabolites and glucosinolate (GSL) biosynthesis genes is being observed in *D. erucoides* than *B. juncea*. The higher accumulation of defense-related GSL compounds was also reported in *D. erucoides* in comparison to *B. juncea* when infected with *A. brassicae* (Choudhury et al., 2022). The study is important in highlighting a few possible candidate genes for engineering defense responses of susceptible mustard cultivars against *Alternaria* blight (Choudhury et al., 2022). It becomes a herculean task to screen the successive progenies for the introgressed target trait in the crosses involving *B. juncea* and CWRs. This is because the cross-combination of different ploidy levels of the parent species results in a large amount of heterogeneity of the progenies. Thus, more area, time and labor inputs are required for screening of the progenies. The screening process can be facilitated by development of molecular markers which are closely associated with the target trait. Recently, Choudhury et al. (2023) developed sequence-tagged sites (STS) markers closely associated with the introgressed target trait i.e. *Alternaria* blight resistance by resequencing of a resistant and a susceptible line of BC2F7 generation of a cross between *B. juncea* and *D. erucoides.*

It is evident from the above cited literature that robust screening and scoring of *CWRs-A. brassicae* pathogen-host interactions, for the identification of reliable R-gene sources in the *Brassica* wild allies is needed. Further studies on generation of symmetric and stable somatic hybrids between *B. juncea* and wild allies through successful bridging of ploidy gaps would be helpful in successful introgression of *Alterneria* blight resistance into cultivated *B. juncea* varieties. Improving knowledge about elicitors and effectors for *A. brassicae* would also be important for devising successful disease management strategies for *Alternaria* blight disease in Indian mustard.

## White rust resistance

White rust is another major disease of Indian mustard. It is caused by biotrophic oomycete fungal pathogen *Albugo candida* (Kamoun et al., 2015). White rust is characterized by appearance of white to pale-colored pustules containing zoospores on the abaxial surface of leaves, stems and inflorescence. Therefore, the disease is sometimes also termed as white blister rust (Holub et al., 1995). Chlorosis is also observed over adaxial leaf surfaces. Systemic infection may cause stagehead leading to loss of seed formation (Verma et al., 1975; Chand et al., 2022). In *B. juncea*, 23-89% yield losses were reported by white rust infection (Lakra and Saharan, 1989; Chand et al., 2022).

Due to the persisting racial variation of *A. candida*, the known genes which offer resistance against this pathogen are often ineffective (Mehta et al., 2023). It is being suggested that among the wild relatives, *B. tournefortii*, could be utilized in breeding programmes for introgression of tolerance/resistance to white rust disease (Kumar, 2015). Vasupalli et al. (2017) developed *B. juncea* introgression lines using resistant *D. erucoides* as donor and susceptible *B. juncea* as the recurrent parent. Firstly, to bridge the ploidy gap between donor and the recurrent parent, *B. rapa* was used as bridging species. *D. erucoides* × *B. rapa* hybrid was developed by embryo rescue, and amphiploids were developed by colchicine doubling. This amphidiploid ‘*eru-rapa*’ was crossed reciprocally with *B. juncea*. After backcrossing with *B. juncea*, the BC1F1 progenies obtained were further backcrossed. The BC_2_F_1_ progeny was selfed and further progenies progressed through selfing (Bhat et al., 2006; Vasupalli et al., 2017). Mehta et al. (2023) utilized these ILs for studying their resistance characteristics against *A. candida*. Among these ILs, ERJ 39, ERJ 12, and ERJ 15, have shown resistance when inoculated with multiple isolates of the pathogen, *A. candida* at cotyledonary leaf, true leaf, and adult plant stages. While, ERJ 108, ERJ 157, ERJ 159, ERJ 13, and ERJ 32 exhibited resistance against single isolates of *A. candida* (Mehta et al., 2023). Thus, these ILs could be useful sources of genetic resistance against *A. candida*.

In India, consistent *B. juncea* germplasm exploration and pre-breeding efforts, followed by rigorous screening of germplasm and breeding lines at multi locations, both at the uniform disease nurseries (UDN) and national disease nurseries (NDN) levels under the All India Crop Improvement Programme on Rapeseed-Mustard (AICRP-RM) has led to the identification of more than twenty five white rust resistance (WRR) donors among *B. juncea* germplasm which are being registered at ICAR-NBPGR, New Delhi (Kumar et al., 2019). These white rust resistant donors are being utilized in different WRR breeding programmes of *B. juncea* in India. Currently, CWRs could be a less sought after solution for resistance against white rust due to availability of WRR germplasm of *B. juncea*.

## Aphid resistance

Among the various insect species which infest *Brassica* species, mustard aphid, *Lipaphis erysimi* Kalt. is reported to cause major losses to *Brassica* yield (Patel et al., 2004; Patel et al., 2019). Fast multiplication of these aphids along with phloem sap-feeding mechanism affects plant growth, and leads to poor seed formation with low oil content (Rohilla et al., 2004; Rana, 2005). Across different agro-climatic conditions, and severity of infection and crop growth stage, *L. erysimi* is known to cause damage ranging from 35.4-91.3% to mustard crop (Ahuja et al., 2010; Kular and Kumar, 2011). Use of synthetic insecticides and other chemical control measures for checking the spread of this pest causes serious environmental pollution which imposes health hazards (Abbaszadeh et al., 2011). This caters the need for robust screening of plant genomic sources for successful introgression of traits for aphid resistance into Indian mustard.

During initial attempts, embryo rescue had been deployed for developing inter-specific hybrids between *B. juncea* and *B. tournefortii*. One of the partially fertile hybrids obtained out of these crosses exhibited tolerance to aphid attack (Kumar et al., 2001). A study pertaining to screening of weedy and wild allies for resistance to *L. erysimi*, identified B. *fruticulosa* and *B. montana* as most promising CWRs for this trait (Kumar et al., 2011). *B. fruticulosa* was crossed with *B. juncea* as a donor parent to synthesize the amphiploid AD-4. Resistance to *L. erysimi*, was exhibited by both *B. juncea* introgression and amphiploid lines. High accumulation of lectins was proposed to be the resistance mechanism in *B. fruticulosa and* amphiploid AD-4 (Kumar et al., 2011). In further studies, Atri et al. (2012) attempted introgression of aphid resistance from *B. fruticulosa* into *B. juncea*. They deployed the artificially synthesized amphiploid, AD-4 (*B. fruticulosa × B. rapa* var. brown *sarson*), as a bridge to transfer resistant traits from *B*. *fruticulosa* to *B. juncea* (Atri et al., 2012). Normal meiosis and pollen grain fertility was exhibited by many introgression lines which carried the euploid chromosome number (2n = 36). Intriguing transcriptomic studies on *B. juncea* infestation with a natural host (*L. erysimi*) and non-natural host cowpea aphid (*A. craccivora*) has highlighted the mechanistic differences of defense response activation in *B. juncea* by both species (Duhlian et al., 2020). This has shed light on transcriptional reprogramming of the host with respect to the genes regulating oxidative homeostasis, defense hormones and secondary metabolite pathways in *B. juncea* during aphid invasion (Duhlian et al., 2020).

Due to scarcity of high yielding aphid-resistant cultivars, the menace of aphid attack in Indian mustard is mainly managed by spray of harmful insecticides (Sachan and Purwar, 2007). This further necessitates the need for a genetic solution for resistance against aphid attack. For reducing the economic losses posed by aphids, CWRs could prove to be a sought-after genetic solution. Therefore, primarily, it is important to mine the genetic resistance resources among CWRs of Indian mustard. We have tabulated recent research on the utilization of crop wild relatives for the introgression of biotic and abiotic stress resistance into *B. juncea* (**Table 2**).

**Table 2:** Recent research on the utilization of crop wild relatives for the introgression of biotic and abiotic stress resistance into *B. juncea*.

S. No.	Biotic/abiotic stressors	Studies related to use of CWRs for identification of stress resistance traits and their introgression into *B. juncea*	References
1.	***Sclerotinia* stem rot (SSR)**	Marker-trait associations (MTAs) in *B. juncea*-*B. fruticulosa* introgression lines (ILs) for SSR-resistance	Rana et al. (2017)
		Genotyping by sequencing of *B. juncea*-*B. fruticulosa* ILs for studying MTAs for SSR-resistance	Atri et al. (2019)
		Development of introgression lines of *B. juncea*-*E. cardaminoides* for SSR resistance	Rana et al. (2019)
		Generation of somatic hybrids of *B. juncea* and *S. alba* with resistance against SSR	Kumari et al. (2020a)
2.	***Alternaria* blight**	Introgression of *Alternaria* blight resistance from *D. erucoides* into *B. juncea*	Bhat et al. (2006)
		Generation of *B. juncea*-*S. alba* somatic hybrids with resistance against *Alternaria*	Kumari et al. (2020a)
		Identification of key glucosinolate biosynthesis genes for *Alternaria* blight resistance in *D. erucoides*	Choudhury et al. (2023)
3.	**White rust**	Development of *D. erucoides*-*B. juncea* ILs	Bhat et al. (2006); Vasupalli et al. (2017)
		Studying the resistance responses of *D. erucoides*-*B. juncea* ILs against different *Albugo candida* isolates	Mehta et al. (2023)
**4.**	**Aphids**	Screening of *B. fruticulosa* and *B. montana* for resistance against aphid attack	Kumar et al. (2011)
		Development of *B. fruticulosa*-*B. juncea* ILs for introgression of aphid resistance	Atri et al. (2012)
5.	**Drought stress**	Transcriptome studies on genes involved in drought tolerance responses in *S. alba*	Kashyap et al. (2023)
		Evaluation of drought tolerance characteristics in *B. fruticulosa*	Dong et al. (2012)
6.	**Heat stress**	*B. juncea*-*S. alba* ILs showed successful seed set under high temperature stress	Kumari et al. (2018)
		Evaluation of heat stress tolerance in *B. tournefortii*	Kashyap et al. (2023)

## Use of CWRs for introgression of abiotic stress resistance traits into *B. juncea*

Environmental factors, like high temperatures, water scarcity and soil salinity cause significant losses to crop yields (Yadav et al., 2020). Constant efforts are being made to explore abiotic stress regulation responses in *B. juncea*, which mostly are multi-genetically regulated. Due to changes in global environmental conditions, there is an urgent need to develop water use efficient and heat stress tolerant Indian mustard genotypes (Singh and Choudhary, 2003; Gill et al., 2012; Singh et al., 2021b). Genetic diversity available among CWRs could be a useful resource for introgression of these abiotic stress resilience traits into high yielding cultivars of Indian mustard (Kapazoglou et al., 2023; Kashyap et al., 2023).

## Drought stress

According to Shekhawat et al. (2012), *B. juncea* is cultivated in nearly 85% of total harvested area, out of which, 37% is mainly rain-fed. Thus, the crop produces and quality of Indian mustard is intensively affected by drought stress. Impacts of drought stress are more severe in the eastern and western parts of India which are more prone to drought (Chauhan et al., 2007). Water stress during stem elongation and pod development stages affects pod setting, thus affecting overall yield of mustard. Drought stress causes yield losses ranging from 17-94% in Indian mustard (Akanksha et al., 2020). Introgression of drought tolerance traits into Indian mustard might include transfer of different morpho-physio and biochemical traits which control drought-tolerance characteristics (Singh and Choudhary, 2003). There is an urgent need for the development of water-use-efficient Indian mustard varieties, and CWRs might serve as useful resources for developing ones. Research studies have highlighted the differential regulation of certain genes and transcription factors under drought stress in Indian mustard (Bhardwaj et al., 2015; Wei et al., 2023). This has shed light on the coding transcripts which could be associated with drought tolerance traits in *B. juncea*.

Previous studies report *S. alba*, a wild relative of Indian mustard, to be drought tolerant (Warwick, 1993). Recently, a study involving wild species and U-triangle species of *Brassica* for their potential tolerance to drought during germination and early seedling stage reported *B. fruticulosa* as drought tolerant (Kashyap et al., 2023). Under PEG-induced drought stress conditions, *B. fruticulosa* exhibited increased proline content than the control plants (Kashyap et al., 2023). In another study, the genome-wide transcriptional profiling of *S. alba* leaves under drought and rewatering conditions revealed numerous gene expression changes under such conditions (Dong et al., 2012). Overall, down-regulation of 309 genes and up-regulation of 248 genes was reported under these conditions. Identified differentially expressed genes were shown to be involved in cell division, catalytic and metabolic process functions (Dong et al., 2012). Broad classification highlighted two categories of gene functions in this study, one encoding protective proteins, like, oxidoreductase, and another encoding regulatory proteins, like, transcription factors (Dong et al., 2012). Further, studies might be helpful in detailed revelation of *B. fruticulosa* and *S. alba* genetic factors which control the drought tolerance characteristics in these CWRs.

The potential of somatic hybridization and embryo rescue has not yet been fully utilized for introgression of drought tolerance traits from CWRs into the cultivated high yielding varieties of *B. juncea* because of post-fertilization barriers leading to embryo abortion. In this regard, the potential of *S. alba and B. fruticulosa* reported to exhibit drought resistance traits could be explored in future research programmes.

## High temperature stress

Mostly grown in rabi season, Indian mustard grows well in tropical and sub-tropical regions as winter oilseed crop (Thakur et al., 2020). Temperature growth conditions for optimal germination and seed set ranges between 25°C to 33°C (Wilson et al., 2014). Heat stress affects *B. juncea* growth both at early (germination and seedling stage) and late (flowering and seed ripening stage) stages, resulting in economic losses to mustard production (Sandhu et al., 2019). Crop wild relatives (CWRs) could serve as useful resources for transfer of heat resilience traits into *B. juncea*. Along with some other stress resistant traits, *S. alba* also possesses heat stress tolerance traits. Through protoplast fusion, Kumari et al. (2018) developed hybrids between *B. juncea* and *S. alba* for transferring of genes responsible for *A. Brassicae* and heat stress tolerance. They were successful in generating somatic hybrids showing resistance against *A. brassicae*, which were able to set seeds at temperatures greater than 38°C, thus, also exhibiting characteristics related to heat tolerance. A recent study conducted by Kashyap et al. (2023) highlights heat stress tolerance characteristics of another wild ally of Indian mustard*, B. tournefortii* (Rawa). When exposed to heat stress, *B. tournefortii* (Rawa) showed maximum percent increase in germination (38.46%). This study highlights heat tolerance capacities of *B. tournefortii* (Rawa) during early stages of growth, i.e. germination (Kashyap et al., 2023).

**Figure 1:**
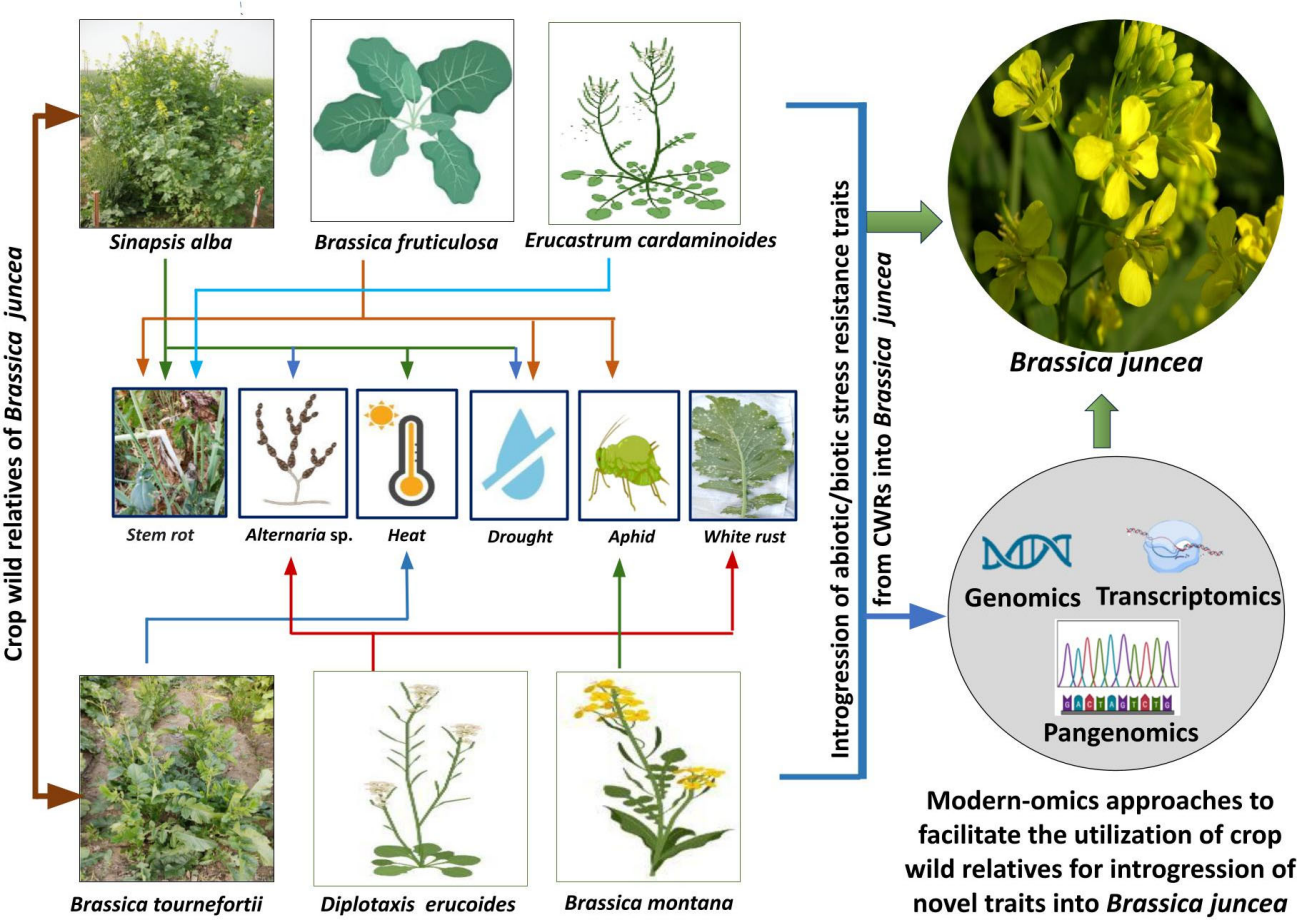
Utilization of specific CWRs for the introgression of biotic/abiotic stress resistance traits into *B*. *juncea* and modern -omics approaches which could be further helpful in assisting successful introgression.

Studies pertaining to utilizing wild allies for introgression of heat stress resilience into Indian mustard are still in infancy. The above surveyed literature suggests that at this stage, it would be beneficial to take up studies concerning evaluation of different *B. juncea* CWRs for their heat tolerance traits. The advances made in transcriptomics and genomics could be utilized for identification of specific genes responsible for regulation of heat stress tolerance in CWRs. This would largely facilitate further research work on introgression of heat stress tolerance traits from CWRs into *B. juncea.* **Figure 1** illustrates the utilization of specific CWRs for the introgression of biotic/abiotic stress resistance traits into *B. juncea*.

## Various methods and molecular techniques to facilitate successful introgression of traits from CWRs into *B. juncea*

### Somatic hybridization and embryo rescue

Several wild plant species exhibit sexual incompatibility with crop species, thereby rendering the genes found in wild forms inaccessible (Branca and Cartea, 2010). Reproductive compatibility in *Brassica* species is complex, with intricate partial barriers between many of them (Han et al., 2021). This is difficult due to the pre- and post-fertilization barriers and abortion of hybrid embryos obtained after inter-specific crosses. Due to the advancements made in *in vitro* culture and embryo rescue, some success has been obtained in development of interspecific hybrids (Pen et al., 2018; Akmal, 2021). The embryo culture technique has proved to be a powerful tool for overcoming post-fertilization barriers between distantly related species, enabling successful hybridization (Pen et al., 2018; Akmal, 2021).

**Figure 2:**
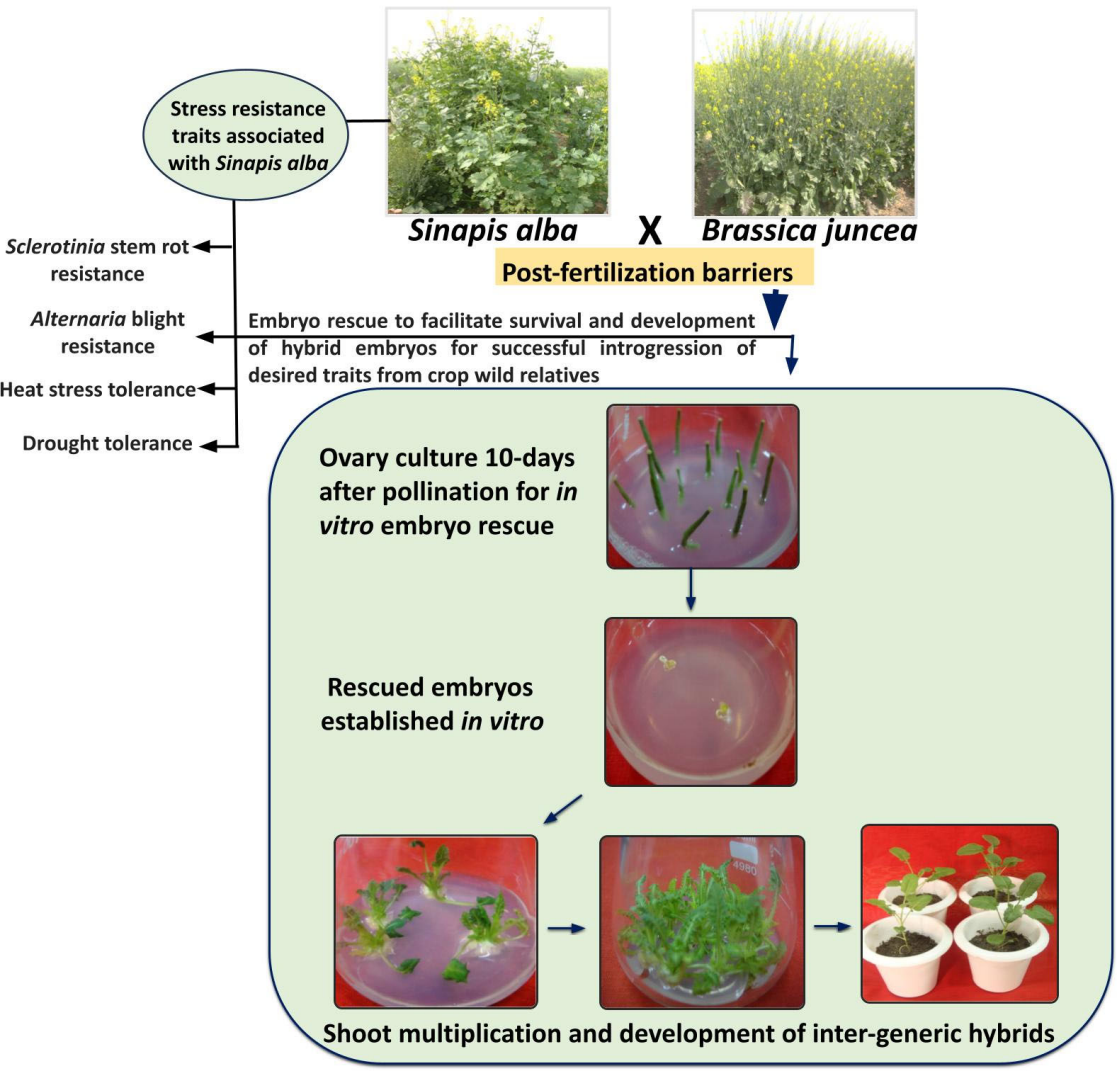
Abiotic and biotic stress tolerance traits associated with *S. alba* and protocol for generation of inter-generic hybrids of *B*. *juncea* and *S. alba* using ovary culture and embryo rescue technique.

By continuous refinement of organ culture protocols, gradual progress is being achieved in obtaining stable inter-generic hybrids utilizing *in vitro* embryo culture methods (Akmal, 2021). Interspecific hybrid production through embryo rescue is often deployed in *Brassica* to overcome post-fertilization barriers. The success of this technique relies on the developmental stage of the rescued embryos (Katche et al., 2019). It is being suggested that embryo rescue can be deployed as early as 10 to 30 days after pollination in *Brassica* (Quazi, 1988; Inomata, 1993; Yuping and Wojciechowski, 2000). Different groups reported use of embryo culture and ovary culture for production of interspecific hybrids between *B. juncea* and *B. campestris*, and between *B. juncea* and *B. napus* (Mohapatra and Bajaj, 1988; Zhang et al., 2001; Zhang et al., 2003). Literature discussed in the above sections of this review reports successful utilization of embryo rescue for the transfer of useful traits related to climate resilience from CWRs into *B. juncea* (Kumar et al., 2001; Bhat et al., 2006; Vasupalli et al., 2017). However, till yet, the efforts to produce interspecific hybrids between CWRs and cultivated *Brassica* are still in infancy. The success of embryo rescue is largely dependent upon the stage of embryos, composition of the medium, and on the genotype to some extent (Katche et al., 2019; Ripa et al., 2020). Therefore, further standardization of protocols pertaining to these parameters would be largely helpful in obtaining successful inter-specific hybrids between *B. juncea* and CWRs. **Figure 2** depicts the stress-resistance traits associated with *S. alba* and protocol for generation of inter-generic hybrids of *B. juncea* and *S. alba* using ovary culture and embryo rescue technique.

### Use of CWRs for cytoplasmic male sterility system development for hybrid breeding in *B. juncea*

Strong hybrid vigor is shown by Brassicaceae crops which have long been subject to F_1_ hybrid breeding. Cytoplasmic male sterility (CMS) system is often relied upon for successful F_1_ seed production in Brassicaceae (Yamagishi and Bhat, 2014). When cytoplasm of an alien species is present in the genetic background of another species, this confers CMS (Yamagishi and Bhat, 2014). During earlier attempts, *B. juncea* var. ‘Pusa Bold’, with the cytoplasmic background of *Diplotaxis siifolia* was developed through wide hybridization (Rao et al., 1994). The cytoplasmic substitution was achieved by repeated backcross of synthetic alloploid (*D. siifolia* x *B. juncea*) with *B. juncea* (Rao et al., 1994). Later, another CMS system was developed in *B. juncea* following repeated backcrossing of the somatic hybrid *Moricandia arvensis* (2n = 28, MM) + *B. juncea* (2n = 36, AABB), carrying mitochondria and chloroplasts from *M. arvensis*, to *B. juncea* (Prakash et al., 1998). Subsequently, Malik et al. (1999) developed two CMS lines using the bridgecross hybrids (*D. erucoides* × *B. campestris*) × *B. juncea* and (*D. berthautii* × *B. campestris*) × *B. juncea*. These were backcrossed with *B. juncea* (Malik et al., 1999). Further, an improved cytoplasmic male sterile (CMS) system of *B. juncea* having cytoplasm of the wild species *D. berthautii* was developed by Bhat et al. (2008). The fertility restorers of *M. arvensis* and *D. catholica*-based alloplasmic CMS systems of *B. juncea* were deployed for restoring the male fertility in these developed CMS lines (Bhat et al., 2008).

Among the various CMS systems adopted in breeding programmes of *Brassica* crops (Ogura, 1968; Yamagishi and Bhat, 2014; Katche et al., 2019), Ogura CMS system has been the most widely utilized. In this system, alien cytoplasm has been obtained by crossing *B. napus* to *Japanese radish* (*Raphanus sativus*) (Ogura, 1968). Complete pollen abortion, ease of transfer and high progeny sterility rates, reaching 100%, are the various advantages associated with Ogura CMS system (Ren et al., 2022). This system has been extensively deployed in *B. napus, B. juncea* and *B. oleracea* (Yamagishi and Bhat, 2014). Wild relatives are often utilized for the development of male sterile lines to help hybrid production (Katche et al., 2019). Among the various CMS systems developed in *B. juncea*, one CMS system is developed by incorporation of cytoplasm from wild relative *B. fruticulosa* (Atri et al., 2016). This has been achieved by the backcross substitution of *B. juncea* (2n = 36; AABB) nucleus into the cytoplasm of *B. fruticulosa*. Complete and stable male sterility was observed in the *B. juncea* genotypes which developed rudimentary anthers with sterile pollen grains and no observed changes in other vegetative parts (Atri et al., 2016). Successful *B. fruticulosa* introgression was documented in at least three chromosomes of *B. juncea*. Further, F_1_, F_2_ and test cross progenies were developed by hybridization between cytoplasmic male sterile and fertility restoring genotypes (Atri et al., 2016). The CMS-fertility-restorer system developed utilizing wild relatives of *B. juncea* holds significant potential for hybrid seed production in Indian mustard. Detailed studies on evolutionary relationships of CMS and fertility restorer genes would be helpful in establishing efficient F_1_ hybrid breeding systems in Brassicaceae crops.

### Modern -omics tools and techniques

In the context of global climate change, CWRs could be promising genetic resources of abiotic and biotic stress resistance (Kapazoglou et al., 2023). In the recent past, successful breeding efforts have certainly helped in introgression of stress-resistant traits from CWRs into *B. juncea.* However, genetic bottlenecks have always been an issue (Kumar et al., 2011; Atri et al., 2012; Kapazoglou et al., 2023). The primary step for the introduction of any novel gene into a crop involves successful identification of reliable genetic resources associated with these traits. Future research on transcriptomic profiling (RNA-seq) of CWRs alone, and in comparison with *B. juncea* cultivars would unravel the molecular mechanisms specifically associated with stress tolerance in CWRs. This would be helpful in the generation of a comparative transcriptomic-profile of the genomic regions, specific stress-responsive genes and biochemical pathways associated with stress tolerance in CWRs and cultivated counterparts of *B. juncea*. Whole genome sequencing and super-pangenomic research would help in gaining an insight into the total gene pool and the available genetic diversity of CWRs of *B. juncea* (Khan et al., 2020). It would also facilitate development of molecular markers which could be utilized for targeted *B. juncea* breeding programs. Additionally, it would be helpful in enhancing our existing knowledge on the complex genetic relationships and genomic introgression events which have occurred between CWRs and *B. juncea*.

In the recent past, cutting-edge genomics research has led to accurate functional characterization of various genes. It has also helped in elucidating the molecular regulators which may underlie biotic/abiotic stress tolerance (Tirnaz et al., 2022; Kapazoglou et al., 2023). Modern genomics research could be helpful in elucidation of novel gene functions in wild relatives of *B. juncea*. In this regard, CWR mutant and over-expression lines could be deployed for assessment of accurate gene functions associated with oil and fatty acid composition and quality. Till today, any research on this aspect is largely lacking. The identified novel-genes could be used for genetic transformation of *B. juncea* with the aim of bio-fortification of oil quality. Bohra et al. (2022) have reviewed in detail how the genetic potential of CWRs can be reaped for producing future crops.

## Conclusions and future perspectives

Due to reduced stringency for the selection of yield-related traits, CWRs of *B. juncea* are valuable sources of resistance against various biotic and abiotic stresses. As reviewed, different crop wild relatives viz. *B. fruticulosa*, *B. tournefortii, B. montana, D. erucoides, S. alba* and *E. cardaminoides* have been used as donors for introgression of stress resistance traits into cultivated *B. juncea.* However, researchers need to design a pragmatic approach, case-by-case basis for resolving the pre- and post-fertilization barriers for successful transfer of traits of interest. Taking into consideration the ploidy levels of the donor and recipient species, some researchers have opted for utilization of bridge species like *B. rapa* (diploid species). While, others deployed protoplast fusion and embryo rescue for development of inter-generic hybrids of *B. juncea* and CWRs with improved stress resistance. For the stabilization of newly introduced traits many generations of recurrent parent back-crossing and open-pollination is further required in these hybrids. Therefore, in every generation, it becomes a herculean task to screen the desired hybrid plants expressing the trait of interest by crop genotyping and phenotyping.

In the past few years, the advances made in omics technologies have discovered novel information on the molecular regulators of biotic and abiotic stress tolerance in different crop plants. These -omics advances have yet not been fully utilized for mining of genes and molecular regulators of stress tolerance in *B. juncea* CWRs. While, use of transcriptomics would enhance our knowledge on differential regulation of various genes under stress, further genomics studies would be helpful in functional characterization of these genes, followed by their targeted transfer into elite high yielding varieties of *B. juncea* using biotechnological tools. Further, whole genome sequencing of CWRs and pangenomics would increase our understanding of how genotypic and phenotypic diversity was shaped during domestication/selection processes from CWRs to *B. juncea*. This would also be helpful in solving the challenges related to breeding and conservation of genetic resources. The whole genome sequencing of CWRs would pave the way for identification of the genes governing resistance to a particular trait and thus suitable molecular markers/candidate-gene specific markers may be deployed for selection of the desired plants, ensuring the trait introgression.

## Author contributions

SV: Conceptualization, Resources, Writing – review & editing, Writing – original draft. ND: Writing – original draft, Writing – review & editing. KS: Writing – review & editing. NP: Writing – review & editing. LS: Writing – review & editing. DS: Writing – review & editing. DR: Writing – review & editing. KT: Writing – review & editing. DV: Writing – review & editing. AT: Writing – review & editing, Conceptualization, Resources, Writing – original draft.
